# Editorial: Plant Responses to the Dark Scenario

**DOI:** 10.3389/fpls.2021.688053

**Published:** 2021-05-28

**Authors:** Péter Poór, Attila Ördög, Chentao Lin, M. Iqbal R. Khan

**Affiliations:** ^1^Department of Plant Biology, University of Szeged, Szeged, Hungary; ^2^University of California, Los Angeles, Los Angeles, CA, United States; ^3^Department of Botany, Jamia Hamdard University, New Delhi, India

**Keywords:** circadian, diurnal, germination, senescence, shade avoidance, stress

## Introduction

Light is one of the most important abiotic factors, which is essential for optimal plant growth and development (Hua, 2013). Thanks to the rhythmic changes in light-dark phases on the Earth, the circadian clock has been evolved in the living organism to synchronize internal regulatory processes (Lu et al., 2017). Although most of the scientific data are coming from the light phase of plant life, the importance of the dark phase is also very significant (Ballaré, 2014). The aim of this Research Topic was to get an insight into new findings/opinions to improve the understanding of plant responses to dark. The topic includes original research and review articles which have been grouped based on four categories.

## Germination, Growth And Development in The Dark

Generally, a plant's life begins in the dark, when germination is started in the soil. Under this condition, skotomorphogenesis or etiolated growth is characterized by hypocotyl elongation, apical hook formation and appressed cotyledons (Gommers and Monte, 2018).

Rovira et al. identified MISREGULATED IN DARK9 (MIDA9) as a phytochrome interacting factor (PIF)-repressed gene in the dark which is necessary for hook development. MIDA9 encodes the type 2C phosphatase PP2C.D1 which negatively regulates SMALL AUXIN UP RNA (SAUR)-mediated cell elongation. Authors found that PP2C.D1 is required immediately after germination to form the hook. Based on genetic analyses of the PP2C.D family, they described the role of PP2C.D2, PP2C.D5 and PP2C.D1. Besides, they suggested a possible interaction between PP2C.D1 and ethylene in hook formation under darkness.

Other processes of the plant's growth and development in the dark were reviewed by Deepika et al.. The article summarizes dark-mediated changes in plant hormones' regulation of signaling complex CONSTITUTIVE PHOTOMORPHOGENIC/DE-ETIOLATED/FUSCA (COP/DET/FUS) and PIFs that affects plant developmental events such as hook development, elongated hypocotyls, photoperiodic flowering, shortened roots, and plastid development.

## Dark-Induced Senescence and Ripening Physiology

The lack of light caused by shading of the leaves leads to rapid senescence (Liebsch and Keech, 2016). The review of Paluch-Lubawa et al. discussed the effects of dark-induced leaf senescence (DILS) in barley. They focused on the importance of chloroplasts, the role of Rubisco, morphological changes, transcriptomic differences, and autophagy in DILS. They identified two steps of DILS program: cessation of photosynthesis accompanied by loss of chlorophyll and disintegration of chloroplasts, followed by the terminal phase, marked by the initiation of cell death and autophagy.

Fruit ripening and seed development are highly dependent on light and active photosynthesis. Farci et al. investigated the effects of dark on the growth of capsules of *Nicotiana tabacum* during the whole post-anthesis period. They found that etiolated capsules showed reduced photosynthetic rates but their seeds had an increased mass and volume, an alteration in gibberellins and abscisic acid, and reduced dormancy compared to the uncovered plants showing the effects of dark in fruit physiology.

## Dark-Induced Senescence and Ripening Physiology

The temperature variations between day and night affect plant growth and interact with the time lag to the light cycle. Effects of the time lag on the circadian rhythm and growth were investigated by Masuda et al.. Firstly, the rhythm of the clock gene CIRCADIAN CLOCK ASSOCIATION 1 (CCA1) was measured and shown to be modulated by the time lag with heating every day but not cooling. Seedling growth was also dependent on the time lag of the heating but not on the cooling cycle. These results provide a method to estimate the appropriate combination of light-dark and temperature cycles.

However, seedlings growth is mediated by the regulation of phytohormones depending on the day and night temperatures. Ohtaka et al. found that a positive DIF (Difference in day and night temperature) treatment, higher temperatures promoted stem elongation. This was accompanied by the upregulation of gibberellin and indole-3-acetic acid synthesis genes. Negative DIF inhibited stem elongation, which was accompanied by the downregulation of gibberellin and indole-3-acetic acid synthesis genes. Thus, DIF treatment can be employed as an effective technique in ornamental horticulture.

Effect of diurnal temperature amplitude on photosynthesis/respiration and growth are less-investigated in C4 crops. Sunoj et al. determined the effects of optimal (27°C) and high (35°C) mean temperatures with three different amplitudes (2, 10, and 18°C) in sorghum. Comparing the high mean daytime and night-time temperatures to optimum values, soluble sugars, starch, leaf area and biomass were reduced, while night respiration was increased but photosynthesis did not change. This revealed the importance of understanding the dark phase mechanisms, especially effects of temperature on night respiration.

Understanding the regulation of water homeostasis and stomatal regulation has great importance under global warming. Qin et al. demonstrated how shade and drought impact the residual leaf conductance (*g*_*res*_) in oak species. In addition, the role of nocturnal conductance (*g*_*n*_) and its relation with *g*_*res*_ were also determined. The authors showed that in closely related species the effect of radiation and drought were interactive in the case of *g*_*res*_, which correlated positively with leaf non-structural carbohydrates and negatively with leaf nitrogen content.

Overnight chilling stress causes a serious decrease in yield in many cultivation areas worldwide. Wu et al. examined the effects of exogenous calcium in the alleviation of nocturnal chilling-induced inhibition of photosynthesis in peanut. They found that foliar calcium application enhanced plant growth and alleviated the nocturnal chilling-dependent feedback limitation of photosynthesis by facilitating cyclic electron flow and decreasing the proton gradient across thylakoid membranes.

## Plant Defense Responses in The Dark

Plant defense responses against pathogens are mostly well-studied processes but the impact of dark signals are less-investigated. The review of Iqbal et al. discussed the effects of dark/light on plant-pathogen interactions, focusing on the role of reactive oxygen species, phytohormones, and transcription factors.

This Research Topic covers the current knowledge of plant responses to the dark scenario. Contributors gave a deep insight into understanding the effects of dark on growth and development, as well as defense responses of plants. These results can help not only plant scientist but also breeders and promote new perspectives in the research of plant responses under darkness.

## Author Contributions

PP, AÖ, CL, and MK: writing—review and editing. All authors contributed to the article and approved the submitted version.

## Conflict of Interest

The authors declare that the research was conducted in the absence of any commercial or financial relationships that could be construed as a potential conflict of interest.

## References

[B1] Ballaré C. L. (2014). Light regulation of plant defense. Annu. Rev. Plant Biol. 65, 335–363. 10.1146/annurev-arplant-050213-040145 24471835

[B2] Gommers C. M. Monte E. (2018). Seedling establishment: a dimmer switch-regulated process between dark and light signaling. Plant Physiol. 176, 1061–1074. 10.1104/pp.17.01460 29217596 PMC5813566

[B3] Hua J. (2013). Modulation of plant immunity by light, circadian rhythm, and temperature. Curr. Opin. Plant Biol. 16, 406–413. 10.1016/j.pbi.2013.06.017 23856082

[B4] Liebsch D. Keech O. (2016). Dark-induced leaf senescence: new insights into a complex light-dependent regulatory pathway. New Phytol. 212, 563–570. 10.1111/nph.14217 27716940

[B5] Lu H. McClung C. R. Zhang C. (2017). Tick tock: circadian regulation of plant innate immunity. Annu. Rev. Phytopathol. 55, 287–311. 10.1146/annurev-phyto-080516-035451 28590878

